# Supplementary figures and images for: Clinical and serological predictors of post COVID-19 condition–findings from a Canadian prospective cohort study
Source: Front Public Health. 2024 May 9;12:1276391. doi: 10.3389/fpubh.2024.1276391 (PMC11111987; doi:10.3389/fpubh.2024.1276391)

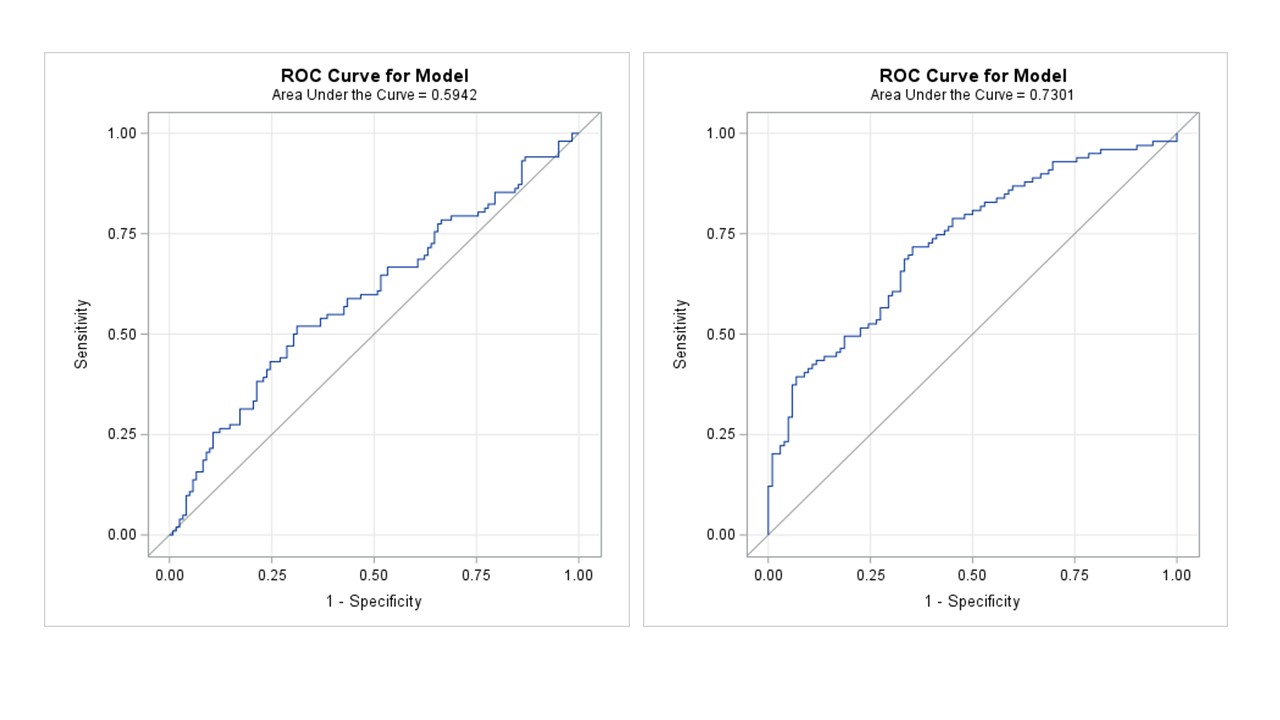

Supplement: Supplementary file 1 [file Image_1.JPEG]

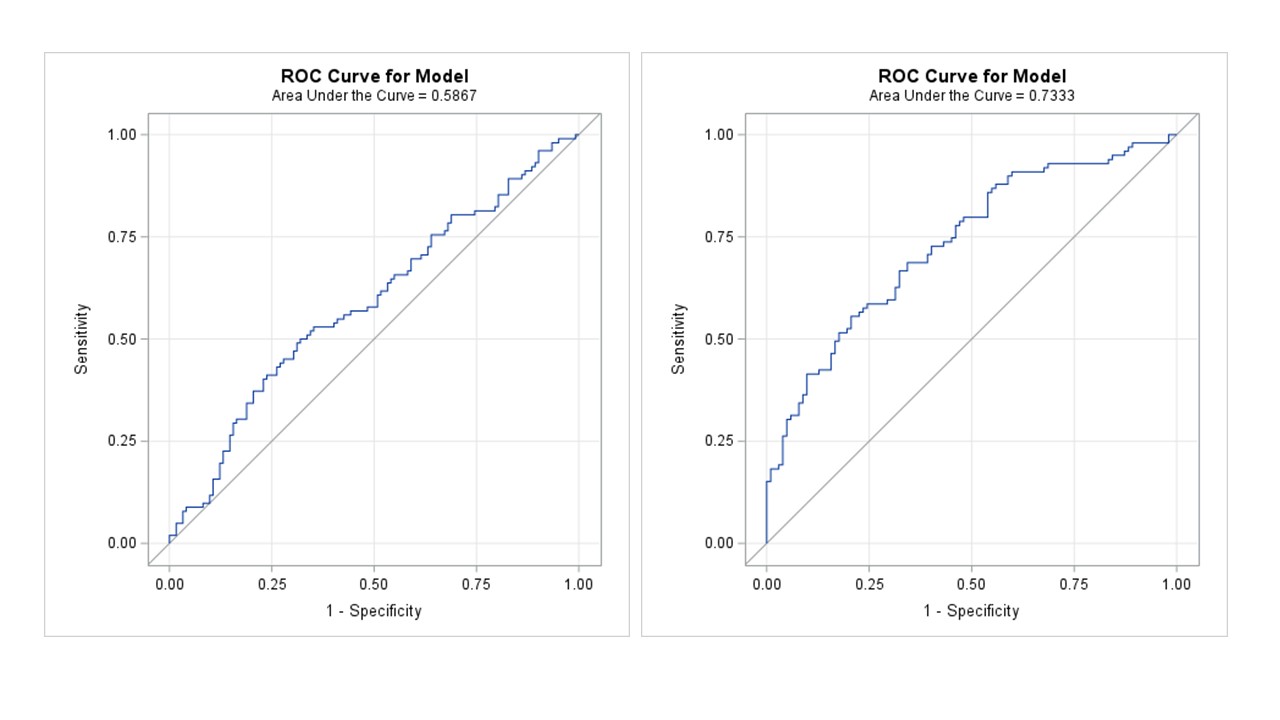

Supplement: Supplementary file 2 [file Image_2.JPEG]

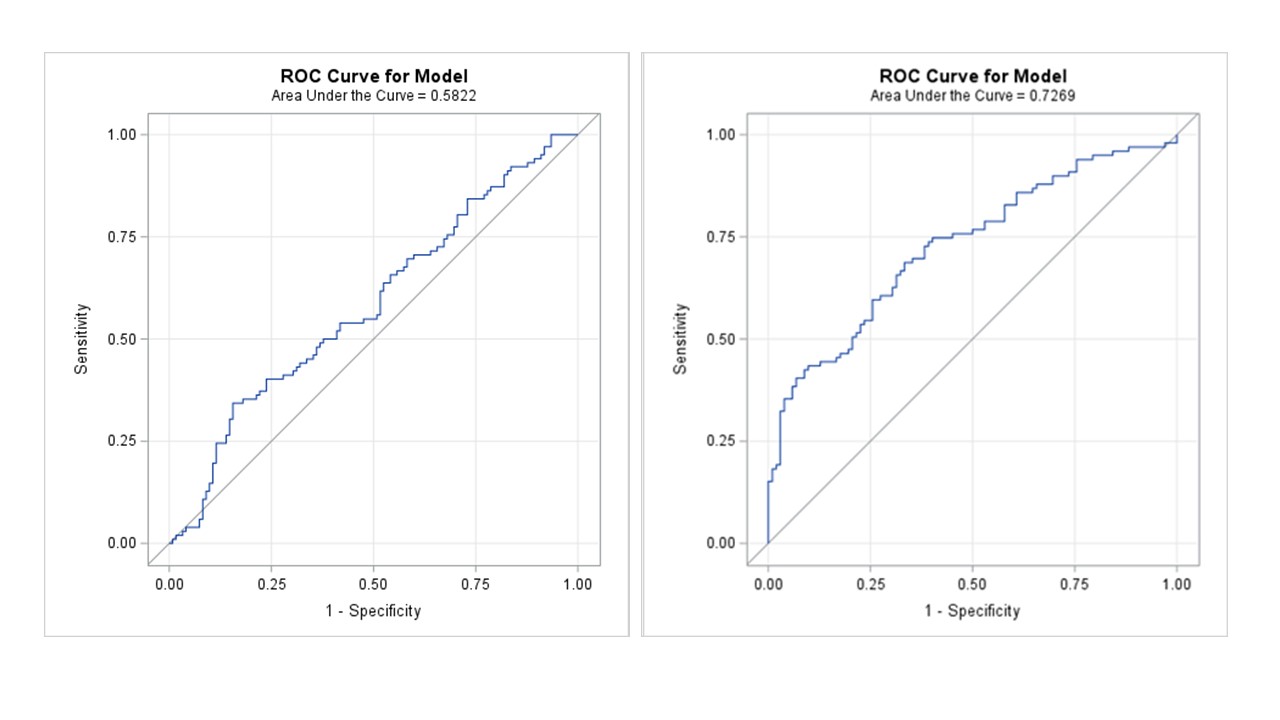

Supplement: Supplementary file 3 [file Image_3.JPEG]

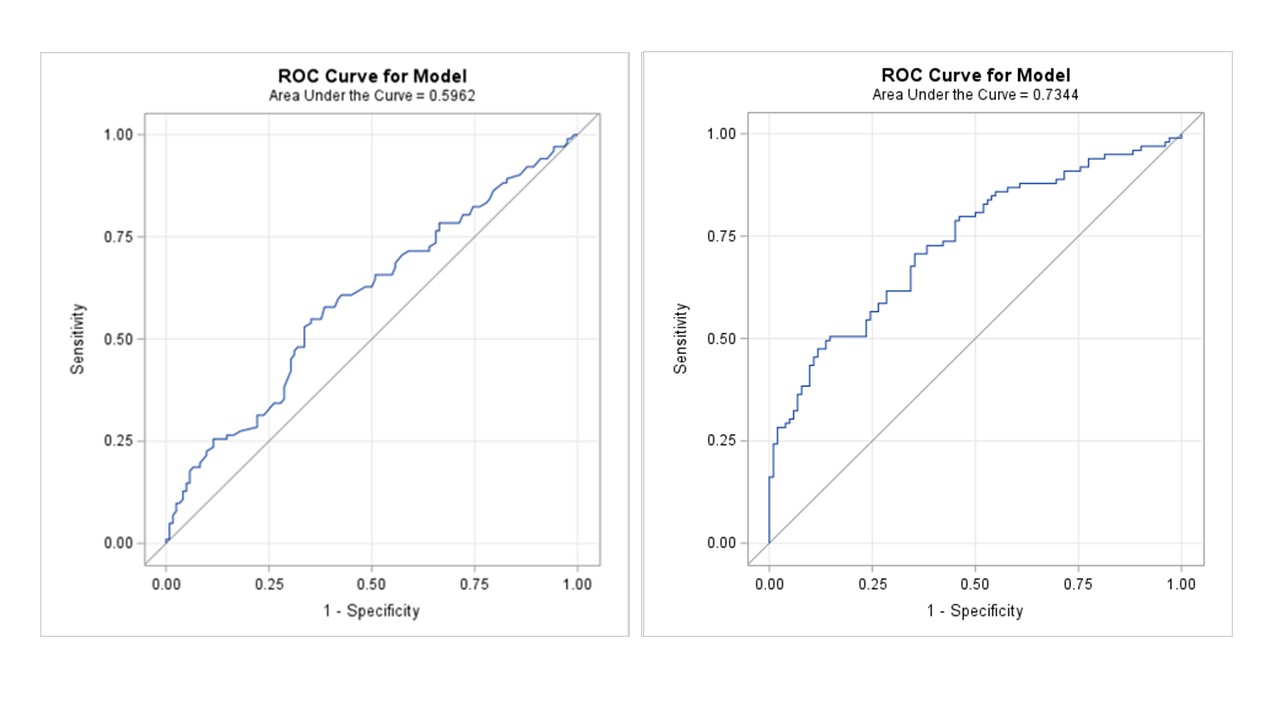

Supplement: Supplementary file 4 [file Image_4.JPEG]
